# Moxibustion Treatment for Knee Osteoarthritis: A Multi-Centre, Non-Blinded, Randomised Controlled Trial on the Effectiveness and Safety of the Moxibustion Treatment versus Usual Care in Knee Osteoarthritis Patients

**DOI:** 10.1371/journal.pone.0101973

**Published:** 2014-07-25

**Authors:** Tae-Hun Kim, Kun Hyung Kim, Jung Won Kang, MinHee Lee, Kyung-Won Kang, Jung Eun Kim, Joo-Hee Kim, Seunghoon Lee, Mi-Suk Shin, So-Young Jung, Ae-Ran Kim, Hyo-Ju Park, Hee-Jung Jung, Ho Sueb Song, Hyeong Jun Kim, Jin-Bong Choi, Kwon Eui Hong, Sun-Mi Choi

**Affiliations:** 1 Korea Institute of Oriental Medicine, Dae-Jeon, South Korea; 2 College of Korean medicine, Gachon University, Seongnam, South Korea; 3 Department of Acupuncture & Moxibustion, Korean medicine hospital, Pusan National University, Yangsan, South Korea; 4 Department of Acupuncture & Moxibustion, College of Korean Medicine, Kyung-Hee University, Seoul, South Korea; 5 Kyungwon University Incheon Gill Oriental Medical Hospital, Incheon, South Korea; 6 Semyung University Jecheon Oriental Medical Hospital, Jecheon, South Korea; 7 Dongshin University Gwangju Oriental Hospital, Gwangju, South Korea; 8 Department of Acupuncture and Moxibustion, Daejeon University, Daejeon, South Korea; University of Ottawa, Canada

## Abstract

**Introduction:**

This study tested the effectiveness of moxibustion on pain and function in chronic knee osteoarthritis (KOA) and evaluated safety.

**Methods:**

A multi-centre, non-blinded, parallel-group, randomised controlled trial compared moxibustion with usual care (UC) in KOA. 212 South Korean patients aged 40–70 were recruited from 2011–12, stratified by mild (Kellgren/Lawrence scale grades 0/1) and moderate-severe KOA (grades 2/3/4), and randomly allocated to moxibustion or UC for four weeks. Moxibustion involved burning mugwort devices over acupuncture and Ashi points in affected knee(s). UC was allowed. Korean Western Ontario and McMaster Universities Questionnaire (K-WOMAC), Short Form 36 Health Survey (SF-36v2), Beck Depression Inventory (BDI), physical performance test, pain numeric rating scale (NRS) and adverse events were evaluated at 5 and 13 weeks. K-WOMAC global score at 5 weeks was the primary outcome.

**Results:**

102 patients (73 mild, 29 moderate-severe) were allocated to moxibustion, 110 (77 mild, 33 moderate-severe) to UC. K-WOMAC global score (moxibustion 25.42+/−SD 19.26, UC 33.60+/−17.91, p<0.01, effect size  = 0.0477), NRS (moxibustion 44.77+/−22.73, UC 56.23+/−17.71, p<0.01, effect size  = 0.0073) and timed-stand test (moxibustion 24.79+/−9.76, UC 25.24+/−8.84, p = 0.0486, effect size  = 0.0021) were improved by moxibustion at 5 weeks. The primary outcome improved for mild but not moderate-severe KOA. At 13 weeks, moxibustion significantly improved the K-WOMAC global score and NRS. Moxibustion improved SF-36 physical component summary (p = 0.0299), bodily pain (p = 0.0003), physical functioning (p = 0.0025) and social functioning (p = 0.0418) at 5 weeks, with no difference in mental component summary at 5 and 13 weeks. BDI showed no difference (p = 0.34) at 5 weeks. After 1158 moxibustion treatments, 121 adverse events included first (n = 6) and second degree (n = 113) burns, pruritus and fatigue (n = 2).

**Conclusions:**

Moxibustion may improve pain, function and quality of life in KOA patients, but adverse events are common. Limitations included no sham control or blinding.

**Trial Registration:**

Clinical Research Information Service (CRIS) KCT0000130

## Introduction

Knee osteoarthritis (KOA) is a common disease which is related to the chronic degenerative changes of knee joint structures [1]. The prevalence of KOA is expected to increase in future because of rising life expectancy [1] and increasing obesity population [2]. Pharmacological treatments are usually recommended for the relief of pain but severe adverse effects related to drug therapy are suggested to be a significant limitation for use [3]. In this point, to discover and to test effectiveness and safety of non-pharmacological interventions is necessary for the vulnerable patients who need long term treatment for KOA.

Moxibustion is a representative non-drug intervention in East Asian traditional medicine. Generally, moxibustion is a method of direct or indirect acupuncture-point stimulation using burned dried mugwort. Although moxibustion is not well known in European countries, it may have been used there in the distant past; several soot marks were found on the body of Ötzi the ‘Tyrolean Ice Man’, half of which were coincident with classic acupuncture points [4].

Moxibustion has been used in clinical practice for conditions such as rheumatic diseases, digestive dysfunction (e.g. dyspepsia, diarrhoea and constipation), and gyneco-obstetric problems (i.e., hot flush and breech presentation of foetus, etc.); however, conclusive evidence on its effectiveness in treating these conditions has yet to be established because few rigorous, full-scale randomised controlled trials have been performed. Moxibustion seems to be effective in the treatment of KOA, but there is currently no rigorous evidence supporting this conclusion [5]. In addition, information on the safety of invasive procedures using burned moxibustion cones is needed.

The primary objective of this study was to test the effect of moxibustion on the pain and function of chronic KOA patients in a pragmatic way. Additionally, the severity and frequency of adverse events (AEs) related to moxibustion treatment were evaluated.

## Methods

This was a multi-centre, randomised controlled, parallel-group, open clinical trial conducted in South Korea. The study protocol was published previously [6]. Participants aged 40 to 70 years with idiopathic osteoarthritis of the knee were recruited through advertisement using local newspapers between June 30, 2011, and January 19, 2012. Idiopathic KOA was diagnosed according to the clinical guidelines of the American College of Rheumatology: the participants should have pain at one or both knees with a daily average of over 40 points on the 0-to-100 numeric rating scale (NRS) and meet at least 3 of the following 6 conditions: age of 50 to 70 years, stiffness within 30 minutes of waking in the morning, crepitus, bony tenderness, bony enlargement or no palpable warmth [7]. Participants with positive rheumatoid factor (RF) in blood chemistry or a history of rheumatoid arthritis, cancer, traumatic injury or significant deformity of the knee, knee-replacement surgery, knee arthroscopy within the last 2 years, steroid injection within the last 3 months or viscosupplement injection and joint-fluid injection within the last 6 months were excluded. Four local research hospitals, namely Oriental Hospital of Daejeon University (Korea Institute of Oriental Medicine), Kyungwon University Incheon Gill Oriental Medical Hospital, Dongshin University Gwangju Oriental Hospital and Semyung University Jecheon Oriental Medicine Hospital, participated in this trial. The protocol for this trial, supporting CONSORT checklist and STRICTA checklist are available as supporting information; see Checklist S1, Checklist S2 and Protocol S1.

Random sequences for allocation were generated with a computer software package (SAS Version 9.1.3, SAS institute. Inc., Cary, NC) by an independent statistician. To avoid casual baseline imbalances in KOA severity, stratification was performed by separate randomisation of each stratum according to the Kellgren/Lawrence scale: both knee x-rays of anterior-posterior and lateral view was obtained from all the patients before allocation. Radiologist read x-rays and graded patient's status based on the Kellgren/Lawrence scale. Participants with grades 0 and 1 were considered as one stratum, and those with grades 2, 3 and 4 were considered as the other stratum. Block size for the randomization was 4 with an allocation ratio of 1: 1. Opaque, sealed envelopes containing serial numbers with a stratification code were used for allocation concealment, and the participants were assigned at the second visit by opening the envelopes in a sequential manner. To reduce the selection bias, the outcome was assessed by individuals who had not participated in the moxibustion treatment. Outcome assessors were intended to be blinded in the study protocol, but outcome assessors were not blinded to the treatment actually, because they became to know about the patients' allocation result from the different visit frequencies in moxibustion group and usual care group as the study went on.

### Ethics statement

The institutional review boards of each participating research centre including Oriental Hospital of Daejeon University, Kyungwon University Incheon Gill Oriental Medical Hospital, Dongshin University Gwangju Oriental Hospital and Semyung University Jecheon Oriental Medicine Hospital reviewed and approved the study protocol before the enrolment of the first patient (2011. 7. 19.). Participants were informed about the moxibustion therapy including moxibustion devices, stimulating methods, intensity and frequency of treatment and possible adverse events. After that, written informed consent was obtained from each participant.

### Interventions

Moxibustion was used for experimental intervention, and a usual care group was used as the comparator. In the moxibustion group, moxibustion therapy on the affected knee(s) was offered at six standard acupuncture points (ST36, ST35, ST34, SP9, Ex-LE04 and SP10), plus up to two points of ‘Ashi’ unilaterally, if needed, three times per week for four weeks. Points were chosen according to the traditional Korean medicine (TKM) literature and to the consensus of four TKM doctors with clinical and research experience. Ashi meant tender points which were not included in classic acupuncture points [8] and were often selected as treatment points of moxibustion for KOA [9], [10]. For patients with pain in both knees, treatments were provided bilaterally. A total of three moxibustion cones were applied indirectly to each point per treatment session. Each burned moxibustion cone was held in place for approximately 5 to 10 minutes and was removed when a patient could no longer tolerate the stimulation. Smokeless, paper devices which had a cylindric shape with a diameter of 1.9 cm and a length of 2.1 cm were used to hold the mugwort for the indirect treatments which means that there is no direct contact between moxibustion and skin (Manina moxibustion, Haitnim Bosung Inc., South Korea). All moxibustions were attached to the skin by adhesive placed on the base of each paper device. Moxibustion was delivered by board-certified KM doctors or postgraduate TKM doctors who had at least 2 years of clinical experience following the standard 6 years of education in KM. To enhance TKM doctor's adherence to the study protocol, all treatment providers were encouraged to attend one-day education program on the moxibustion method. All patients received an educational leaflet containing basic information about KOA such as definition, pathology, current treatment options including drug therapy, supplements and hyaluronic acid or steroid injection and recommendations on the principles of self-exercise, good postures and rules for daily activities avoiding exaggerating symptoms. In addition, participants were instructed in exercises to stretch hamstrings and calf muscles and strengthen muscles related to the function of knee joints by research TKM doctors. The frequency and intensity were not prescribed equally but participants were encouraged to increase them along with the physical fitness.

Each component of usual care was based on previous evidence of benefits in the management of KOA [11]. Co-interventions allowed to both groups in all study periods included surgery, conventional medication, physical therapy, acupuncture, herbal medicine, over-the-counter drugs and other active treatments.

Before allocation, all participants were asked to rate their expectations on a 0-to-9 (higher expectation) numeric scale. The question was as follows: “To what degree do you expect moxibustion to relieve your symptoms?”.

### Outcomes

The primary outcome was measured by the Korean Western Ontario and McMaster Universities Questionnaire (K-WOMAC) global score [12]. This was a Korean-translated validated version of WOMAC which was widely used for the evaluation of knee pain and function related to osteoarthritis [13]. It consisted of 24 questions about knee pain, stiffness and physical function. Individual questions were summed up in three domains each and global score was calculated as well through summing up of these three subscales which ranges 0 to 100 (the worst) [6]. The Short-Form 36 Health Survey (SF-36v2) was assessed for quality of life evaluation [14]. The Beck Depression Inventory (BDI) was used to measure the severity of depression [15]. Physical performance affected by disability related to KOA was evaluated [16], [17]. Three functional tests were assessed for each participant: the timed-stand test, standing-balance test and six-minute walk test [18]. The 7-day average score on the pain-numeric rating scale from 0 (least pain) to 100 (most pain) was assessed. All the outcomes were assessed up to 13 weeks after first visit principally. We used validated Korean-translated tools for all outcome assessment except physical performance test. In our study, unit of analysis were individual patients not affected knees. Patients with pain in one knee and in both knees were included without discrimination. Outcomes for knee pain and function were observed in the knee with more severe symptoms. Blood chemistry including C-reactive protein and erythrocyte sedimentation rate at 5 weeks and cost-effectiveness at 5 and 13 weeks were assessed as secondary outcomes. These results will be published elsewhere in future.

To evaluate safety, we assessed the occurrence of adverse events (AEs) related to moxibustion during the trial. Local and systemic AEs were assessed in every visit. If unexpected responses related to moxibustion occurred, the type, severity and frequency were reported. The severity of each AE was graded 1 (mild) to 3 (severe) according to Spilker's AE classification [19], [20]. Burn wounds were diagnosed as first to third degree [21]. All outcomes were assessed by separate outcome assessors who did not participate in moxibustion treatment. AEs related to usual care treatments were not assessed in this study.

Because there were no field-specific standards for data deposition available for moxibustion study, data related to the study results could not be publicly assessed.

### Statistical analysis

The study sample size was calculated from the unpublished internal data of our pilot-study which was conducted with 40 KOA patients at Korea Institute of Oriental Medicine in 2010. In the pilot-study, only patients with grades over 2 on the Kellgren/Lawrence scale were included at first but we extended inclusion criteria during the study due to a low participation rate. We adopted revised inclusion criteria in this multi-centre trial. The mean difference and pooled standard deviation of the K-WOMAC global score between the moxibustion and usual care groups was estimated to be 15.4 and 6.65, respectively. With a two-sided 5% significance level, 80% power and 20% dropout rate, a total of 212 participants needed to be recruited.

To compare key baseline characteristics between the two groups, the Chi-squared test or Fisher's exact test for categorical data and the t-test or the Wilcoxon rank-sums test for continuous data were conducted after the Kolmogorov–Smirnov test for normality. Age and body mass index which might affect the severity of KOA symptom were not controlled in both groups for all analysis. Most outcomes (i.e., K-WOMAC, pain NRS, BDI, Physical Function Test and SF-36v2) were analysed using analysis of covariance (ANCOVA), with baseline scores and individual research centres as covariates. However, because KOA patients with different disease severity were allocated equally in 2 groups through stratified randomization method based on the Kellgren/Lawrence scale, KOA grade of each participant was not used as covariates. Eta-squared (η^2^) was calculated as an effect-size estimate in the ANCOVA statistic model [22]. The T-scores of each domain for the SF-36v2 assessment were calculated using Health Outcomes Scoring software 4.5 (QualityMetric Incorporated, Lincoln, RI). All statistical analyses were conducted on an intention-to-treat basis at a 95% significance level. The last observation carried forward (LOCF) method was used to input missing data. All statistical analyses were conducted using SAS statistical software. There was no Data Monitoring Committee in this study.

## Results

**Figure 1:**
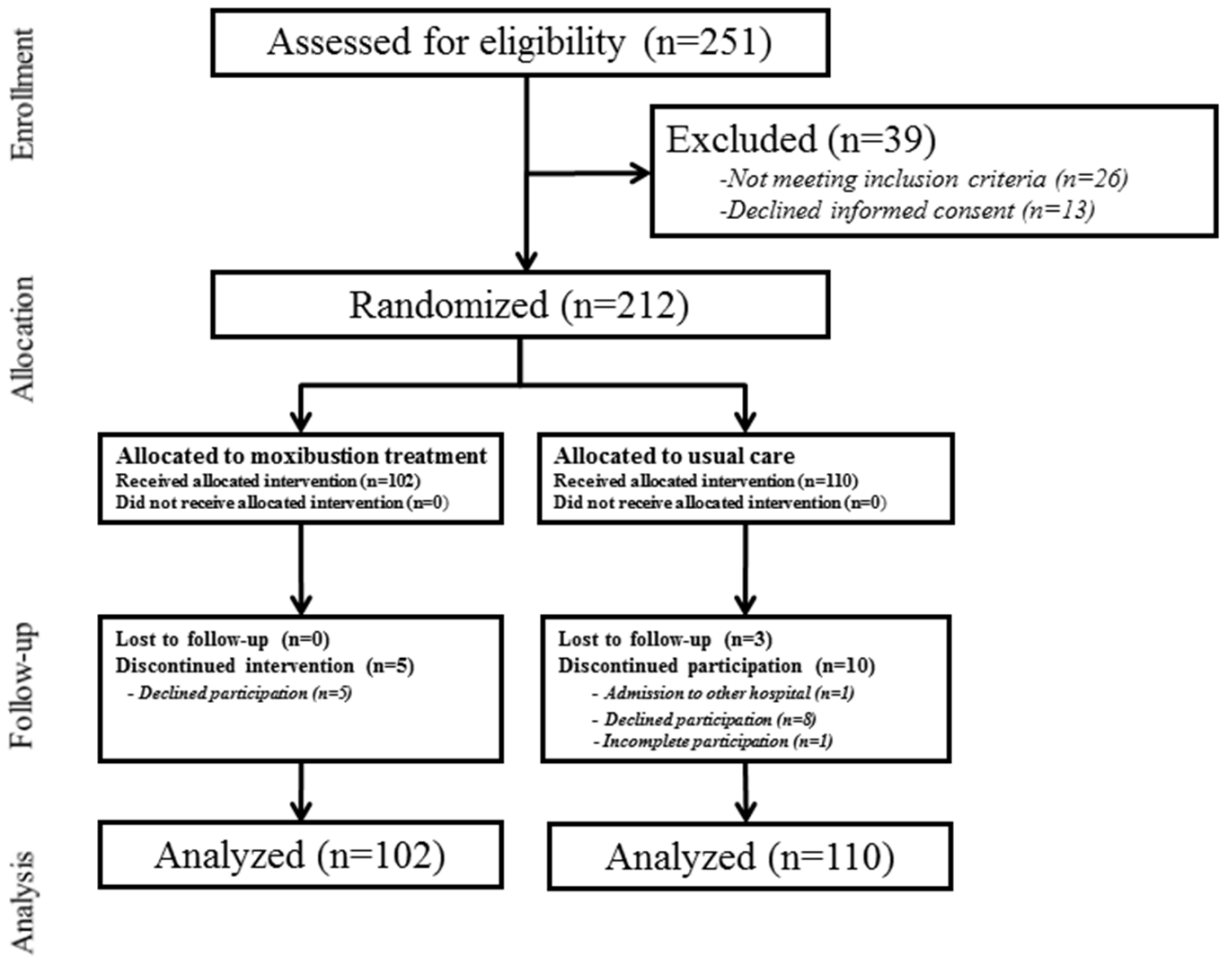
Study flow chart.

A total of 251 participants were recruited, and 212 KOA patients met the inclusion criteria. Incheon, Gwangju and Jecheon hospitals recruited 60 participants each, and Daejeon centre recruited 32 participants. Of these, 102 patients (73 for mild and 29 for moderate to severe KOA) were placed in the moxibustion group and 110 (77 for mild and 33 for moderate to severe KOA) were placed in the usual care group. During the study, 5 participants in the moxibustion group and 9 participants in control group dropped out (figure 1). Five participants in the moxibustion group and eight in the control group were dropped out due to withdrawal of consent. Other reasons for the dropout in control group were admission to other hospital due to other diseases and incomplete participation during the clinical trial.

Median duration of knee pain was 4 years [2 to 6 years] in the moxibustion group and 3 years [1 to 6 years] (interquartile ranges) in the usual care group. According to the Kellgren/Lawrence scale, most participants had mild-to-moderate KOA. Many patients had previously tried physical therapy, acupuncture treatment and glucosamine administration for KOA. Including age and body mass index, all baseline characteristics did not show significant different between groups. In addition, there was no significant difference between the groups in terms of their expectations of the effectiveness of moxibustion (Table 1).

**Table 1:** Demographic Data in the moxibustion and usual care Groups.

Characteristics	Moxibustion group (n = 102)	Usual care group (n = 110)	p-value
Age, year (median [25% to 75% IQR])*	56 [52 to 62]	57 [51 to 62]	0.74
Sex M/F, No.†	17/85	16/94	0.67
Duration of knee pain, year (median [25% to 75% IQR])*	4 [2], [6]	3 [1], [6]	0.28
Body mass index, Kg/m^2^ (mean, standard deviation)‡	24.77, 2.63	24.09, 2.94	0.08
Kellgren/Lawrence scale§			0.3579
Grade 0	24	36	
Grade 1	49	41	
Grade 2	25	29	
Grade 3	4	3	
Grade 4	0	1	
Past experience of surgery for knee osteoarthritis (yes/no)§	2/100	0/110	0.23
Past experience of anti-arthritic medication for knee osteoarthritis (yes/no)†	28/73	37/71	0.31
Past experience of physical therapy for knee osteoarthritis (yes/no)†	53/49	60/50	0.71
Past experience of acupuncture treatment for knee osteoarthritis (yes/no)†	34/67	41/68	0.55
Past experience of intra-articular injection treatment for knee osteoarthritis (yes/no)†	23/78	29/80	0.52
Past experience of glucosamine administration for knee osteoarthritis (yes/no)†	33/68	36/74	0.99
Assessment of expectation on the effectiveness of moxibustion (median [25% to 75% IQR])*	7 [6 to 9]	7 [5 to 8]	0.33

*The Wilcoxon rank sum test was used for statistical analysis.

† The Chi-squared test was used for statistical analysis.

‡ The t-test was used for statistical analysis.

§ Fisher's exact test was used for statistical analysis. IQR: interquartile range.

### K-WOMAC

The global K-WOMAC score (primary outcome) showed significant differences between the 2 groups at 5 weeks (25.42 (SD 19.26) in the moxibustion group and 33.60 (17.91) in the usual care group; p<0.01) and 13 weeks (26.70 (18.82) in the moxibustion group and 34.69 (18.67) in the control group; p<0.01). According to Cohen's benchmark for effect size [22], a small-to-medium effect size was observed at 5 weeks (0.0477) and 13 weeks (0.0518, Table 1).

All subcategories of K-WOMAC showed significant improvement following moxibustion treatment at 5 weeks and 13 weeks. The pain score showed a comparatively large effect size at 5 weeks (0.0532) and 13 weeks (0.0595, Table 2).

**Table 2:** Primary and secondary outcomes at each visit.

	Moxibustion group (n = 102, mean, SD)			Usual care group (n = 110, mean, SD)			P-value		Effect size (η^2^)***	
	Baseline	5 weeks	13 weeks	Baseline	5 weeks	13 weeks	5 weeks	13 weeks	5 weeks	13 weeks
K-WOMAC (mean, SD)*										
Global score (total)	34.16, 16.80	25.42, 19.26	26.70, 18.82	34.15, 18.01	33.60, 17.91	34.69, 18.67	<0.01	<0.01	0.0477	0.0518
*Mild severity (Grade 0 and 1)* **	*32.19, 16.88*	*22.14, 17.74*	*23.53, 16.64*	*30.75, 17.37*	*30.95, 17.58*	*32.08, 18.10*	*<0.01*	*<0.01*		
*Moderate to severe severity (Grade 2,3 and 4)* **	*39.10, 15.81*	*33.69, 20.72*	*34.66, 21.76*	*43.03, 17.25*	*41.06, 17.32*	*41.42, 18.62*	*0.2554*	*0.3021*		
Pain score	6.93, 3.48	5.07, 3.75	5.18, 3.83	7.21, 3.8	7.14, 3.94	7.32, 3,98	<0.01	<0.01	0.0532	0.0595
Stiffness score	2.87, 1.6	2.26, 1.67	2.51, 1.68	3.12, 1.82	2.95, 1.75	3.2, 1.78	0.0043	0.0061	0.0226	0.0267
Function score	24.35, 12.91	18.09, 14.46	19.01, 13.89	23.83, 13.29	23.51, 12.94	24.17, 13.68	<0.01	0.0001	0.0391	0.0412
Pain-NRS (mean, SD)*	57.02, 14.3	44.77, 22.73	40.53, 26.63	57.63, 12.93	56.23, 17.71	54.26, 19.61	<0.01	<0.01	0.0073	0.0075
BDI (mean, SD)*	9.85, 7.11	8.94, 7.15	8.75, 6.95	9.8, 7.2	9.63, 7.13	9.03, 6.44	0.34	0.64	0.0023	0.0005
Physical performance test (mean, SD)*										
Timed-stand test (sec)	27.34, 19.16	24.79, 19.77	22.85, 9.76	26.03, 8.83	25.24, 8.84	25.76, 9.09	0.0486	0.0006	0.0021	0.0307
Standing balance test	3.56, 0.79	3.59, 0.71	3.70, 0.58	3.64, 0.67	3.67, 0.59	3.64, 0.69	0.52	0.26	0.0012	0.0048
Six minute walk test (m/6 min)	493.8, 95.1	486.1, 81.3	489.2, 79.3	480.1, 78.2	481.8, 80.4	479.0, 78.0	0.51	0.68	0.0008	0.0004

*ANCOVA was used for the statistical analysis of changes from baseline between two groups on each outcome at 5 weeks and 13 weeks (covariate: baseline value and participating research centre).

**Grades were evaluated by Kellgren/Lawrence scale.

***Eta-squared (η2) was calculated as an effect-size estimate in the ANCOVA statistic model. K-WOMAC: Korean version of Western Ontario and McMaster Universities Questionnaire; NRS: numeric rating scale; BDI: Beck Depression Inventory.

### Pain NRS

Moxibustion treatment improved the average pain NRS significantly compared with usual care at 5 weeks (44.77 (SD 22.73) with moxibustion and 56.23 (17.71) with usual care, p<0.01) and 13 weeks (40.53 (26.63) with moxibustion and 54.26 (19.61) with usual care, p<0.01, Table 2); however, comparatively small effect sizes were observed at 5 weeks (0.0073) and 13 weeks (0.0075).

### Physical performance test

Moxibustion significantly improved knee function for standing and sitting in a chair (as evaluated by the timed-stand test) compared to usual care at 5 weeks (24.79 (9.76) in the moxibustion group and 25.24 (8.84) in the usual care group; p = 0.0486) and 13 weeks (22.85 (9.76) in the moxibustion group and 25.76 (9.09) in the usual care group; p = 0.0006). No significant improvement was observed in the standing-balance test (p = 0.52 at 5 weeks and p = 0.26 at 13 weeks) or six-minute walk test (p = 0.51 at 5 weeks and p = 0.68 at 13 weeks). All results of the three physical performance tests showed relatively small effect sizes at 5 weeks (0.0021 in the timed-stand test, 0.0012 in the standing-balance test and 0.0008 in six-minute walk test) and 13 weeks (0.0307, 0.0048 and 0.0004 respectively).

### BDI

BDI scores improved in the moxibustion group after treatment, but there was no significant difference between the 2 groups at 5 weeks (8.94 (7.15) with moxibustion and 9.63 (7.13) with usual care; p = 0.34) and 13 weeks (8.75 (6.95) and 9.03 (6.44); p = 0.64). Only small effect sizes were observed in BDI at 5 weeks (0.0023) and 13 weeks (0.0005).

### SF-36

The physical component summary (PCS) showed significant improvement following moxibustion treatment at 5 weeks (p = 0.0299) and at 13 weeks (p = 0.0023), but there was no significant difference between groups in mental component summary (MCS) at 5 weeks (p = 0.2124) and 13 weeks (p = 0.3129). Bodily pain (BP) showed significant improvement following moxibustion both at 5 weeks (p = 0.0003) and 13 weeks (p = 0.005). Physical functioning (PF) and social functioning (SF) also showed better results at 5 weeks (p = 0.0025 in PF and p = 0.0418 in SF), but there was no significant difference between the 2 groups in other domains. Small effects sizes were observed in PCS (0.0147 at 5 weeks and 0.0307 at 13 weeks) and in MCS (0.0034 at 5 weeks and 0.0031 at 13 weeks). Among 8 domains of SF-36, only BP showed small to moderate effect sizes at 5 weeks (0.0437) and 13 weeks (0.0410) and others showed only small effect sizes (Table 3).

**Table 3:** The short-form 36 health survey (SF-36v2) results at each visit.

	Moxibustion group (n = 102, mean, SD)			Usual care group (n = 110, mean, SD)			P-value		Effect size (η^2^)*	
	Baseline	5 weeks	13 weeks	Baseline	5 weeks	13 weeks	5 weeks	13 weeks	5 weeks	13 weeks
Physical Component Summary (PCS)	42.39, 6.81	44.32, 6.5	44.43, 6.39	41.19, 6.92	41.89, 7.25	41.31, 7.33	0.0299	0.0023	0.0147	0.0307
Mental Component Summary (MCS)	49.24, 10.13	50.71, 9.77	48.80, 9.45	49.96, 10.19	49.69, 10.59	50.23, 10.48	0.2124	0.3129	0.0034	0.0031
Physical Functioning (PF)	39.98, 8.00	42.04, 7.64	40.79, 7.90	40.51, 7.34	39.74, 7.86	39.62, 7.45	0.0025	0.1214	0.0269	0.0087
Role-Physical (RP)	44.96, 9.88	45.55, 10.12	45.49, 9.74	45.11, 8.92	46.19, 9.12	44.42, 9.47	0.5640	0.2893	0.0010	0.0042
Bodily pain (BP)	45.48, 7.87	49.16, 8.10	48.68, 7.63	43.07, 7.68	44.18, 8.53	43.80, 9.04	0.0003	0.0005	0.0437	0.0410
General Health (GH)	43.23, 8.13	44.39, 9.12	44.19, 8.56	42.13, 8.99	42.70, 7.97	43.72, 8.61	0.3110	0.7561	0.0029	0.0003
Vitality (VT)	48.35, 10.46	50.12, 10.29	50.41, 10.82	46.55, 9.72	47.39, 10.63	48.30, 10.97	0.1725	0.4290	0.0054	0.0021
Social Functioning (SF)	48.79, 9.43	50.85, 8.18	49.23, 8.61	49.50, 7.91	49.05, 9.22	48.77, 8.35	0.0418	0.4487	0.0121	0.0020
Role-Emotional (RE)	44.63, 11.78	45.55, 11.86	42.92, 12.02	45.75, 10.96	45.47, 11.66	44.84, 11.65	0.6133	0.3688	0.0007	0.0027
Mental Health (MH)	47.56, 10.07	49.66, 10.02	48.12, 9.66	48.28, 11.01	47.99, 10.79	48.82, 11.03	0.0865	0.6976	0.0083	0.0005

ANCOVA was used for the statistical analysis of changes from baseline between two groups on each outcome at 5 weeks and 13 weeks (covariate: baseline value and participating research centre).

*Eta-squared (η2) was calculated as an effect-size estimate in the ANCOVA statistic model.

### Adverse events

One hundred and two participants in the experimental group were subjected to moxibustion for a total of 1,158 treatments, with 121 AEs related to the treatment (10.45%). Among these 102 participants, 48 patients experienced AEs at least once during the treatment periods, and 7 participants experienced AEs more than 5 times. First degree burn wounds occurred 6 times, and second degree burns occurred 113 times. Systemic AEs, including pruritus and fatigue, were seen in 2 participants. When AEs were graded according to the Spilker's AE classification, mild AEs occurred 99 times and moderate AEs occurred 21 times. There was only one severe AE.

## Discussion

A total of 212 patients with mild-to-moderate KOA participated in this trial. Dropout rate in the moxibustion group was about 4.9% and that in control group was 11.8%. From the results of this study, 4 weeks of moxibustion treatment improved the global score and sub-scores (pain, stiffness and function) by K-WOMAC and decreased the pain NRS significantly compared with the usual care control at 5 and 13 weeks. However, BDI did not show significant difference between groups at 5 and 13 weeks. Among all domains of SF-36, PCS and BP at both 5 and 13 weeks and PF at 5 week showed significant improvement with moxibustion treatment compared to the control group. Physical performance for sitting and standing from a chair was significantly improved in the moxibustion group than in the usual care group but there were no significant improvements in standing balance and six minute walk test. Approximately 47% of participants experienced at least one AE (mostly burn wounds); the majority of the AEs were second-degree burns.

Recent systematic reviews suggested that moxibustion might be effective in the treatment of KOA, but the supporting evidence was not conclusive because of limitations including methodological flaws, comparatively small sample sizes, inappropriate outcome assessments and poor reporting of adverse events in the previous clinical trials on moxibustion [5], [23]. In this study, we adopted a rigorous clinical trial design to reduce possible bias. Sequence generation and allocation concealment were conducted appropriately. Although blinding of participants was impossible, separate outcome assessors participated in the outcome assessment to reduce performance bias. To ensure a considerable degree of external validity, 4 local research centres in different regions of South Korea participated in this study. The appropriate sample size was calculated from the results of a previous pilot study: 212 participants constituted a larger sample size than was used in previous moxibustion trials [5]. Apart from simple evaluation of pain intensity and participants' ratings of improvement used in previous studies, we assessed various core outcome domains, including physical (physical performance test) and emotional (BDI) functioning, a disease-specific outcome such as K-WOMAC and quality of life (SF-36) to report the results more completely [24]. In addition to these quantitive outcomes, we also conducted a qualitative research for assessing KOA patients' experiences of moxibustion in the perspective of mixed-methods approach whose results were published elsewhere [25]. These various outcomes can suggest evidence useful to understand the effect of moxibustion treatment in the multidimensional aspects. Previous studies using moxibustion reported comparatively low incidence rates of AEs [26] and reported only minor AEs [5]; however, from the explicit AE assessment of this study, as conducted according to the pre-defined reporting criteria, we found that AEs occurred frequently and that moxibustion could induce moderate-to-severe AEs. These factors contribute to the validity of the results and are a major strong point of this study.

This study has several weak points as well. First, sham moxibustion was not used for this study. Several types of sham moxibustion devices have been invented [27], [28] and some have been used in the clinical trials [29], [30]. However, these types of sham devices cannot apply to those who have experience of moxibustion because they have thick membrane between moxibustion and human skin which blocks heat and chemical discharge from the moxibustion so participants cannot feel appropriate moxibustion stimulation [27], [28]. In addition, current studies with sham moxibustion might have potential bias in blinding the patients: a clinical trial did not suggest any blinding test results at all [29] and another trial showed incomplete blinding successfulness where sensitivity was high in verum moxibustion group but low in sham group[30]. We found that appropriate sham device which was completely inactive physiologically but seemed similar to verum moxibustion, was not available currently. In this sense, we adopted usual care as comparison intervention. Second, moxibustion is used in clinical practice with wide heterogeneity in the original materials, stimulating methods, frequency, duration, selection of points, etc [5]. The original purpose of this study was to evaluate the benefit and harm of moxibustion treatment itself, so we used manufactured moxibustion of standardised quality. In this sense, the moxibustion used in this trial is not fully representative and is only one typical intervention among various moxibustions. Third, moxibustion is currently used only in Asia (not in European countries), and the participants' expectations were high in this study. Non-specific effects of moxibustion may have an important role in symptom management, as is the case for other non-drug interventions such as acupuncture. Thus, the study results must be interpreted in a limited context. Fourth, outcome assessors were not blinded which might introduce detection bias in this study. To reduce bias, separate independent researchers, who did not conduct treatments, participated in the outcome assessments but this could not ensure low risk of bias in the outcome assessment procedure. Fifth, we allowed any types of co-interventions to both groups during the study periods. We expected that the pattern of usage of treatments for KOA would not be changed easily during the comparatively short period if past usage of usual care components in the baseline stage were similar in two groups. However, it could not be an appropriate explanation on the potential bias that free access to various treatment options might not introduce considerable imbalance of additional treatments between the groups. Finally, AEs related to usual care treatments were not evaluated appropriately in this study. We tried to assess the AEs related to moxibustion rigorously but we did not pay enough attention to the usage of usual care interventions and related AEs which might overestimate the frequency of AEs in the moxibustion group.

One thing we need to declare is that we eased the inclusion criteria because a low participation rate was observed in the pilot study when only patients with grades over 2 on the Kellgren/Lawrence scale were recruited. We adopted the clinical criteria of the American College of Rheumatology as the diagnostic criteria for KOA [7], so we included KOA patients regardless of the severity as evaluated via X-ray. Instead, stratified randomisation was conducted to avoid baseline imbalances in KOA severity. As a result, significant improvement of the K-WOMAC global scale in the moxibustion group was observed in the mild-KOA group but not in the moderate-to-severe-KOA group. This result may have originated from the unequal number of participants with mild (n = 146) versus moderate-to-severe KOA (n = 62).

Interestingly, the effect sizes of K-WOMAC pain subscale and SF-36 bodily pain component were similar each other but the effect size of pain-NRS was smaller than those of K-WOMAC pain. The pain-NRS is a valid and easy tool for evaluating pain intensity related to KOA [31] but it also has been criticized for its simplicity which prohibits understanding complexity of patient's pain experience [32]. KOA is a chronic condition and many factors are associated with pain experience of the patients with KOA. In this sense, we assume that K-WOMAC pain subscale and SF-36 bodily pain might reflect more closely to the patient's change of pain-related experience than pain-NRS did and it might introduce huge difference in the effect sizes among the evaluation tools.

In future studies, it may be necessary to recruit only patients with moderate-to-severe KOA to evaluate whether moxibustion is effective only in mild cases. To evaluate the specific effect of moxibustion, standardization of original material and practice procedure for moxibustion is necessary and a proper control intervention including sham moxibustion should be developed and used in clinical trials, although such a control would be difficult to devise. Factors contributing to the effect of moxibustion, including selection of acupuncture points and moxibustion devices, intensity, frequency and duration of treatment and the moxibustion method (i.e., direct or indirect application), should be divided and tested in a separate study to evaluate the individual effects. Finally, the long-term outcome of this treatment must be evaluated.

## Supporting Information

Checklist S1 **A CONSORT checklist.**
(DOC)

Checklist S2 **A STRICTA checklist.**
(DOCX)

Protocol S1 **Published protocol of this study.**
(PDF)
